# ADAM15 correlates with prognosis, immune infiltration and apoptosis in hepatocellular carcinoma

**DOI:** 10.18632/aging.203425

**Published:** 2021-08-23

**Authors:** Jun Hui Xu, Yong Jun Guan, Yi Chao Zhang, Zhen Dong Qiu, Yu Zhou, Chen Chen, Jia Yu, Wei Xing Wang

**Affiliations:** 1 Department of Hepatobiliary Surgery, Renmin Hospital of Wuhan University, Wuhan, China; 2 Central Laboratory, Renmin Hospital of Wuhan University, Wuhan, China; 3 Hubei Key Laboratory of Digestive System Disease, Wuhan, China

**Keywords:** ADAM15, apoptosis, immune infiltration, hepatocellular carcinoma, prognosis

## Abstract

ADAM15 is highly expressed in malignant tumors and is correlated with tumor progression. However, the role of ADAM15 in hepatocellular carcinoma (HCC) remains unclear. In the study, our results indicated that ADAM15 was highly expressed in HCC tissues and cells compared with corresponding tissues and liver cells. Overexpression of ADAM15 was linked to poor prognosis, and was an independent risk factor for HCC prognosis. Besides, analysis of immune infiltration indicated that ADAM15 expression was related to tumor infiltrating lymphocytes based on the TIMER, TISIDB and GEPIA databases. Many immune checkpoint gene expression was associated with ADAM15 expression. Functional enrichment analyses indicated that apoptosis, cell adhesion was enriched. ADAM15 knockdown promoted apoptosis and suppressed proliferation, migration and invasion of liver cancer cells. The findings of western blot showed that ADAM15 knockdown reduced the expression of Bcl-2, Vimentin, N-Cadherin and Snail, and elevated the expression of Bax, E-cadherin and ZO-1. However, overexpression of ADAM15 had the opposite results. Collectively, our findings demonstrated that ADAM15 was connected with poor prognosis of HCC patients, and could be considered as a potential biomarker for the diagnosis and treatment of HCC.

## INTRODUCTION

Hepatocellular carcinoma (HCC) is the major histological type of primary liver cancer, and is a big health care burden in the world [1]. HCC ranks the sixth most common cancer and the third cancer-related deaths globally [1, 2]. Currently, there are many ways for HCC treatment, including surgical resection, local ablation therapy, transplantation, transcatheter arterial chemoembolization, and targeted treatment [3–6], the main treatment for early-stage HCC is hepatectomy. Due to many patients are diagnosed at the late stage of HCC, the majority of them lose the opportunity for radical surgical treatment, the 5-year survival rate of patients with advanced HCC after surgical intervention remains less than 18% [7]. Some studies have shown that abnormal gene expression was associated with the occurrence, development and relapse of HCC [8, 9]. So, it’s urgent for us to explore novel markers for early diagnosis and treatment of HCC.

ADAM15 (also named A disintegrin and metalloproteinase 15) belongs to a family of membrane-bound metzincin metalloproteinase (ADAM family) [10] and locates on chromosome 1q21.3 [11]. ADAM15 contains many substrates which include several key cell regulatory molecules, such as E-cadherin and N-cadherin, Transforming Growth Factor Beta (TGFβ) and EGFR ligands [12]. These cell molecules play an important role in regulating cell adhesion and cellular motility [13]. Adhesion of cancer cells to the epithelial-mesenchymal transition (EMT) via integrins can lead to the increase of ROS, resulting in tumor invasion and metastasis [14]. As one of the catalytically active ADAMs, ADAM15 has been reported to be overexpressed in various malignancies, including breast cancer, prostate cancer and lung cancer [15–17]. Upregulation of ADAM15 expression in breast cancer and prostate cancer has been related to tumor aggressiveness and metastasis [15]. While Toquet et al. reported that downregulation of ADAM15 could promote colon cancer metastasis and correlated with poor prognosis [18].

However, there is no information available about the expression of ADAM15 in HCC tissues and paired normal tissues, and the relationship between ADAM15 expression and the prognosis of HCC patients is still unclear. In the study, we investigated ADAM15 expression in HCC tissues and cells, and dissected the role of ADAM15 in the proliferation, migration, invasion and apoptosis of HCC cells.

## MATERIALS AND METHODS

### Data mining from the public databases

The expression of mRNA-seq and clinicopathological information of 371 HCC patients and 50 noncancerous patients were downloaded from the TCGA database (https://portal.gdc.cancer.gov/). Among them, only 365 tumor samples have both mRNA-seq and clinical survival data. The expression of 115 HCC patients and 52 normal tissues were extracted from the Gene Expression Omnibus GSE76427 and the expression of 81 HCC patients and 80 normal tissues were extracted from the GSE54236 database. All the raw count data were analyzed by the package edgeR of R software, and the differential expression of ADAM15 in HCC samples and adjacent non-tumor samples was identified. |log^2^ (fold change).

(|log2FC|) and adjusted *P*-values were used to assess the significance of differentially expressed genes (DEGs). Meanwhile, |log2FC| > 1 and *P* < 0.05 were set as the cut-off criteria. The Estimate algorithm was performed to estimate the infiltration levels of stromal and immune cells in HCC based on the interpretation of gene expression profiles extracted from the TCGA database [19]. The detailed immune checkpoint genes are listed in the Supplementary Table 1.

### The Kaplan-Meier plotter analysis

The Kaplan Meier plotter can be used to estimate the effect of 54k genes (mRNA, miRNA, protein) on survival in 21 cancer types including breast (*n* = 7,830), ovarian (*n* = 2,190), and liver (*n* = 365) cancer. Sources for the databases include GEO, EGA, and TCGA [20].

### The construction of nomograms

Nomogram models were constructed based on ADAM15 expression by using the “rms” and “survival” packages in R. Then, the consistency between actual and predicted survival was estimated by calibration curves.

### GSEA analysis

Gene Set Enrichment Analysis (GSEA) is a computational method that can be used to estimate the connection between ADAM15 expression (high/low groups) and tumor-related pathways. The detailed method followed the protocol from the Broad Institute Gene Set Enrichment Analysis website [21]. Gene sets database was set “c2.cp.kegg.v6.1symbols.gmt”, the number of permutations was set 1000 times, and a normalized enrichment score (NES) was acquired, then gene sets were significant when *P*-value < 0.05.

### The TISIDB analysis

The TISIDB database (http://cis.hku.hk/TISIDB/) is an integrated repository web portal for analysis of interactions between tumors and the immune system [22], which integrated 4176 records from 2530 publications and reported 988 genes related to anti-tumor immunity. The TISIDB was applied to evaluate the role of ADAM15 in tumor immune interplay.

### The TIMER analysis

Tumor Immune Estimation Resource (TIMER) (http://cistrome.org/TIMER/) is an ideal database that contains 10897 samples across 32 cancer types from TCGA [23]. On the one hand, the connection between ADAM15 expression and tumor purity and immune infiltrating cells was computed by the TIMER database based on a previously published statistical deconvolution method from gene expression profiles. On the other hand, the relationships between ADAM15 expression and immune cell surface markers were calculated. Immune gene surface markers shown in Table 2 were retrieved from the website of R&D Systems (https://www.rndsystems.com/cn/resources/cell-markers/immune-cells). The x-axis delegated ADAM15 gene, and the y-axis represented related marker genes. Scatterplots were used to determine the correlation between ADAM15 expression and each immune gene marker.

### The GEPIA analysis

GEPIA (Gene Expression Profiling Interactive Analysis) (http://gepia.cancer-pku.cn/) is a valuable and highly cited resource for gene expression analysis based on tumor and normal samples from the TCGA and the GTEx databases [24]. GEPIA2021, a standalone extension with multiple deconvolution-based analysis for GEPIA [25]. They deconvolute each sample tool in TCGA/GTEx with the bioinformatics tools CIBERSORT, EPIC and quanTIseq.

### Clinical samples collection

15 HCC specimens and matched noncancerous specimens were collected from HCC patients who underwent liver resection in Renmin Hospital of Wuhan University (Wuhan, China) from October 2019 through December 2020. The collected tissue specimens were stored at –80°C for subsequent use. All patients signed informed consent before surgery treatment, and the study was approved by the Ethics Committee of Wuhan University.

### Cell culture and transfection

HCC cells (LM3, Huh7, HepG2 and SMMC-7721 cells) were cultured in DMEM (Servicebio) or RPMI 1640 medium (Servicebio) supplemented with 10% fetal bovine serum and 1% penicillin and streptomycin (Servicebio). Small interfering (si) RNAs si-ADAM15 and overexpression of ADAM15 plasmid were obtained from miaolingbio.Inc, Wuhan, China. The sequences of siRNAs and overexpression plasmid are listed in Supplementary Table 2. HepG2 and SMMC-7721 cells were cultured in 6-well plates (5 × 10^5^/well) and transfected with 2.4 ug siRNA and overexpression plasmid using Attractene Transfection Reagent (Cat.No. 301005, QIAGEN, China).

### Construction of ADAM15 plasmid and transient transfection

The ADAM15 overexpression plasmid pCAG-ADAM15 (human), PEF1-ADAM15 (human), PEnCMV-ADAM15 (human) were obtained from miaolingbio.Inc, Wuhan, China. The overexpression plasmid PEnCMV-ADAM15 (human) was used to transfect HepG2 and SMMC-7721 cells. HepG2 and SMMC-7721 cells were cultured in 6-well plates (5 × 10^5^/well) and transfected with 2.4 ug si-RNA3 or PEnCMV using Attractene Transfection Reagent. Then the transfected cells were performed for further experiments.

### Reverse transcription-quantitative polymerase chain reaction

Total RNA was isolated using Trizol reagent (Invitrogen, USA) following the manufacturer’s protocol. Total RNA (2 ug) was performed to synthesize cDNA, which served as a template for the amplification of ADAM15 and GAPDH (as a housekeeping gene). Quantitative real-time PCR was performed using a SYBR Premix Ex Taq™ II kit (Takara, Dalian, China) on the BIO-RAD Real-time PCR system with 7500 software. The primer sequences for ADAM15 were 5′-3′ (forward) GCCTCAAAAAAGGTGCTTCAGAC and 5′-3′ (reverse) GTTCTCCAAAGTGTGTCCCTCA. GAPDH forward, 5′-3′ GGAAGCTTGTCATCAATGGAAATC and reverse 3′-5′ TGATGACCCTTTTGGCTCCC. The relative expression of ADAM15 was calculated and normalized using the 2^−ΔΔCt^ method.

### Western blot

First of all, the tissues or cell samples were lysed in RIPA buffer with a protease and phosphatase inhibitor mixture (Beyotime Biotechnology, Shanghai, China). Then, using the BCA assay (Beyotime Biotechnology, Shanghai, China) to analyze protein concentrations. Secondly, Thirty micrograms of each protein sample were separated using 12% SDS-PAGE and transferred to nitrocellulose membranes (BD, USA). Then membranes were disposed with 5% (w/v) non-fat milk for 1 hour at room temperature. Thirdly, membranes were incubated with primary antibodies overnight at 4°C. Finally, blots were incubated with horseradish peroxidase-linked secondary antibodies (LI-COR, Cat no. 926-32211, 1:10000 dilution) for an additional 1 hour. Subsequently, the enhanced chemiluminescence system was used to visualize immunoreactive bands. Each experiment was performed in triplicate and protein expression levels were relative to GAPDH. Antibodies used included Anti-ADAM15 mouse monoclonal antibody (1:200; cat. no. sc-365752; Santa Cruz Biotechnology, Inc.), anti-E-cadherin rabbit monoclonal antibody (1:1000; cat. no. ab40772; Abcam), anti-N-cadherin rabbit monoclonal antibody (1:1000; cat. no. ab76011; Abcam), anti-Vimentin rabbit monoclonal antibody (1:1000; cat. no. ab92547; Abcam), anti-Snail rabbit monoclonal antibody (1:1000; cat. no. ab216347; Abcam), anti-ZO-1 rabbit monoclonal antibody (1:1000; cat. no. ab276131; Abcam), anti-Bax rabbit polyclonal antibody (1:1000; cat. no. GB11007; Servicebio), anti-Bcl-2 rabbit polyclonal antibody (1:800; cat. no. AF0060; Beyotime Biotechnology).

### Immunohistochemical staining (IHC)

The experimental method of IHC were processed with our previous methods [26]. Firstly, the sections were baked in the oven, then deparaffinized in xylene and hydrated in ethanol with different concentration gradients. Secondly, antigen repair was performed after PBS washing. Thirdly, endogenous peroxidase blocker was added to the sections at room temperature for 10 min. Fourthly, the sections were incubated overnight at 4°C with primary antibody. Then DAB chromogen was added, hematoxylin was redyed and neutral gum was sealed. The primary antibodies are listed below, Anti-ADAM15 mouse monoclonal antibody (1:100; cat. no. sc-365752; Santa Cruz Biotechnology, Inc.). The sections were observed under light microscopy, and five randomized microscopic views of 200-fold magnification of each section were observed and scored.

### Cell proliferation assay

The method of Cell Counting kit-8 (CCK8) (cat, CKO4-500T; Dojindo Molecular Technologies, Inc.) was performed to determine the proliferation ability of HCC cells following the manufacturer’s instructions. Firstly, the HepG2 and SMMC-7721 cells were plated in 96-well plates (2 × 10^3^ cells/well). Then, HepG2 and SMMC-7721 cells were transfected with siADAM15 or overexpression plasmid PEnCMV-ADAM15. The CCK8 solution (10 ul) was added to each well for 1–4 h at 37°C and absorbance was recorded at 450 nm after 24 hours. Each experiment was performed in triplicate.

### Wound-healing assay

Wound-healing assays were performed to analyze the migration area or distance of HCC cells. HepG2 and SMMC-7721 cells were seeded in a 6-well plate and transfected with siRNA3 or PEnCMV until 90% confluency was reached. A 200 ul pipette tip was performed to create an artificial wound. Then, the cells were washed three times with PBS, and replaced with a serum-free medium. Cell migration was observed using a light microscope (magnification, ×40). The distance or area between the edges of the wound was calculated by Image J 1.51j8 (NIH, USA).

### Cell invasion assay

Invasion experiments were used to assess the invasive capacity of HCC cells. The transfected HepG2 and SMMC-7721 cells were collected, resuspended in 200 ul serum-free medium, and 5 × 10^4^ cells were seeded into 24-well upper Transwell chamber (8 μm pore size) with Matrigel (BD, USA). The lower part of the chamber was full of a medium containing 20% FBS. Invasion culture periods were about 24 h. Cells on the top surface of the filters were wiped off using a cotton swab. The cells on the lower surface of the filter were fixed with 4% paraformaldehyde and stained with 0.1% crystal violet at 37°C for 1 h. The numbers of invading cells were counted using a light microscope with ×200 magnification.

### Flow cytometry analysis

After transfection for 72 h, HCC cells were collected and washed three times with cold PBS solution. According to the instructions, HCC cells were resuspended with 500 ul Annexin V binding buffer with adding 5 ul Annexin V-FITC and 5 ul PI. Following incubation at room temperature for 30 min, and flow cytometer was performed to analyze apoptosis.

### Statistical analysis

Student’s *t*-test or one-way ANOVA with Turkey’s post hoc test in SPSS 23.0 software (IBM Corps.). Pearson’s Chi-square test was utilized to compare the expression level of ADAM15 among different clinicopathological groups. Then, ROC curve analysis was performed to verify the diagnostic value of ADAM15 in HCC. The level of ADAM15 expression was separated into two groups (high/low groups) via X-tile software (version 3.6.1) based on the state of HCC dead or recurrence [27]. The best cutoff value was automatically set based on the maximum *χ*^2^ value, and survival analysis was performed according to the above cutoff value. Then, *P*-value was also computed. The immune scores and stromal scores were separated into low and high groups according to the median value. Survival analysis for overall survival (OS) and recurrence-free survival (RFS) both in the high ADAM15 expression group and low ADAM15 expression group was performed by the Kaplan-Meier method and log-rank test. A Cox proportional hazards regression was used to estimate the independent prognostic risk factors based on OS or RFS, both *P*-value and hazard ratios (HR) with 95% confidence intervals (CI) were computed for different variables. The relationship between ADAM15 mRNA expression and immune cell infiltration was accessed by the TIMER, TISIDB and GEPIA databases. The correlation of immune checkpoint genes with ADAM15 expression was performed by R. *F*-test (one-way ANOVA) was utilized to identify the expression of 11 ROS-related genes in immune cells between tumor tissues and normal tissues. SPSS 23.0 software (IBM Corps.), GraphPad Prism 8.0.2 (GraphPad Software, Inc.) and R (version 3.6.1) were used for statistical analyses. *P*-value < 0.05 was considered to be statistically significant.

### Ethics approval and consent to participate

The study was approved by the Ethics Committee of Wuhan University and the consent was approved by participates.

## RESULTS

### The mRNA and protein expression levels of ADAM15 in HCC tissues and paired noncancerous tissues

**Figure 1:**
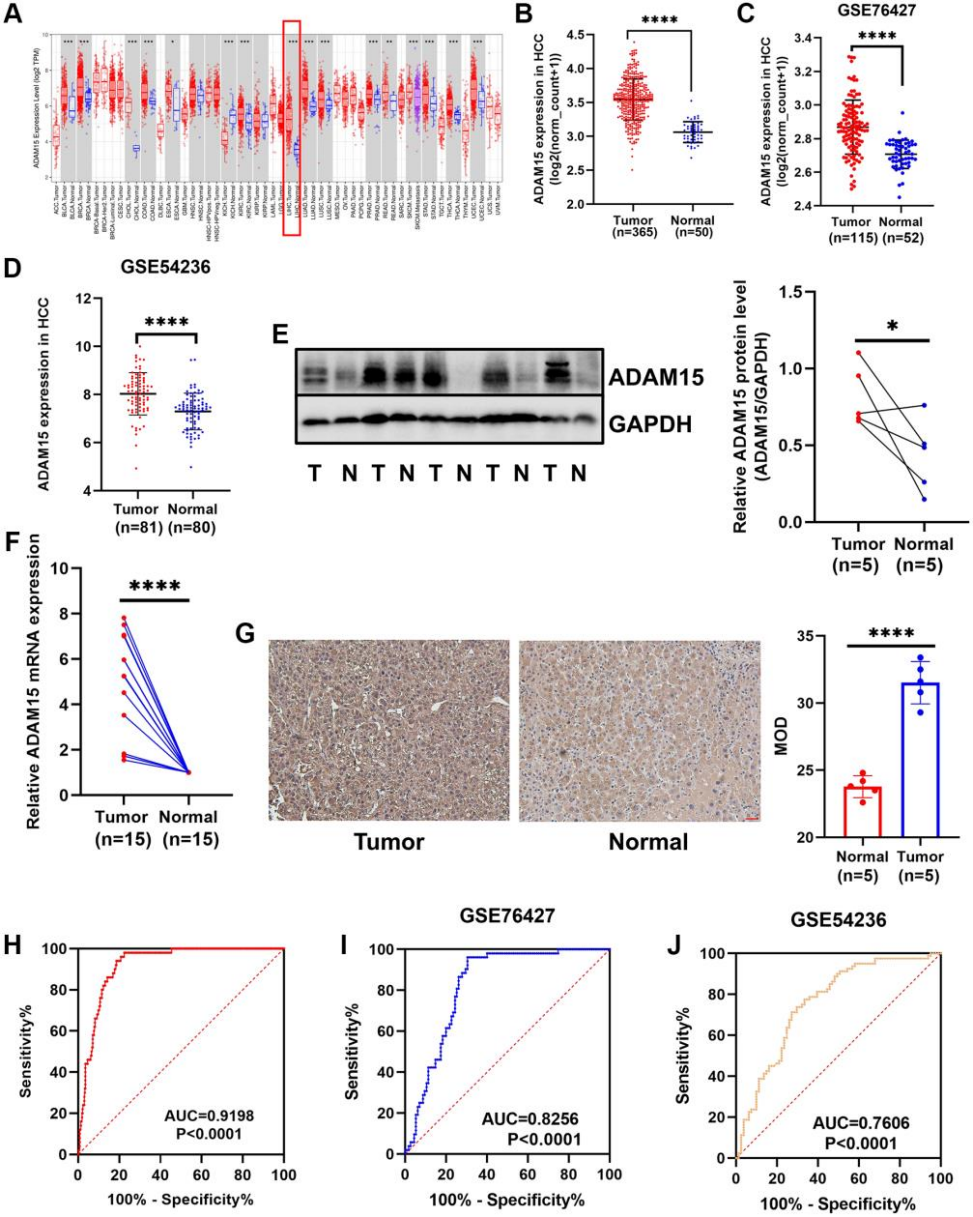
**Different expression of ADAM15 in HCC tissues and corresponding noncancerous tissues.** (**A**) ADAM15 expression in pan-cancer. (**B**) ADAM15 expression was up-regulated in HCC tissues compared with paired noncancerous tissues by the TCGA database. (**C**, **D**) The level of ADAM15 expression was higher in HCC tissues than that in matched noncancerous tissues by the GEO database (GSE76427 and GSE54236). (**E**) ADAM15 expression in HCC samples and paired noncancerous samples was confirmed by western blot, and higher ADAM15 expression was in HCC tissues compared with matched noncancerous tissues. (**F**) ADAM15 expression in HCC samples and paired noncancerous samples was verified by RT-qPCR, and higher ADAM15 expression was in HCC tissues compared with matched noncancerous tissues. (**G**) ADAM15 expression in HCC samples and paired noncancerous samples was confirmed by IHC, and the value of MOD was higher in HCC tissues compared with matched noncancerous tissues. Magnification, ×200. Scale bar: 50 μm (**H**, **I**, **J**) Validation of diagnostic value of ADAM15 overexpression in HCC based on the TCGA and GEO databases.

We analyzed the mRNA expression data of LIHC in TCGA and found that ADAM15 was highly expressed in liver cancer tissues than that in the matched normal tissues (Figure 1A, 1B). Meanwhile, the above result was verified by the GEO database (GSE76427, GSE54236) (Figure 1C, 1D). We further confirmed that ADAM15 was up-regulated in HCC tissues compared with corresponding noncancerous tissues based on the analysis of RT-qPCR, Western blot and IHC. The analysis of Western blot indicated that ADAM15 expression was higher in HCC tissues (*n* = 5) compared with those in paired normal tissues (*n* = 5) (Figure 1E). The result of RT-qPCR revealed that ADAM15 expression was significantly upregulated in HCC tissues (*n* = 15) than that in the matched noncancerous tissues (*n* = 15) (Figure 1F). Furthermore, the result of IHC also demonstrated that higher ADAM15 expression in HCC tissues (*n* = 5) than that in matched normal tissues (*n* = 5) (Figure 1G). Mean Optical Density (MOD) was served as an indicator for IHC.

### Overexpression of ADAM15 independently predicted poor OS and RFS in HCC

**Figure 2:**
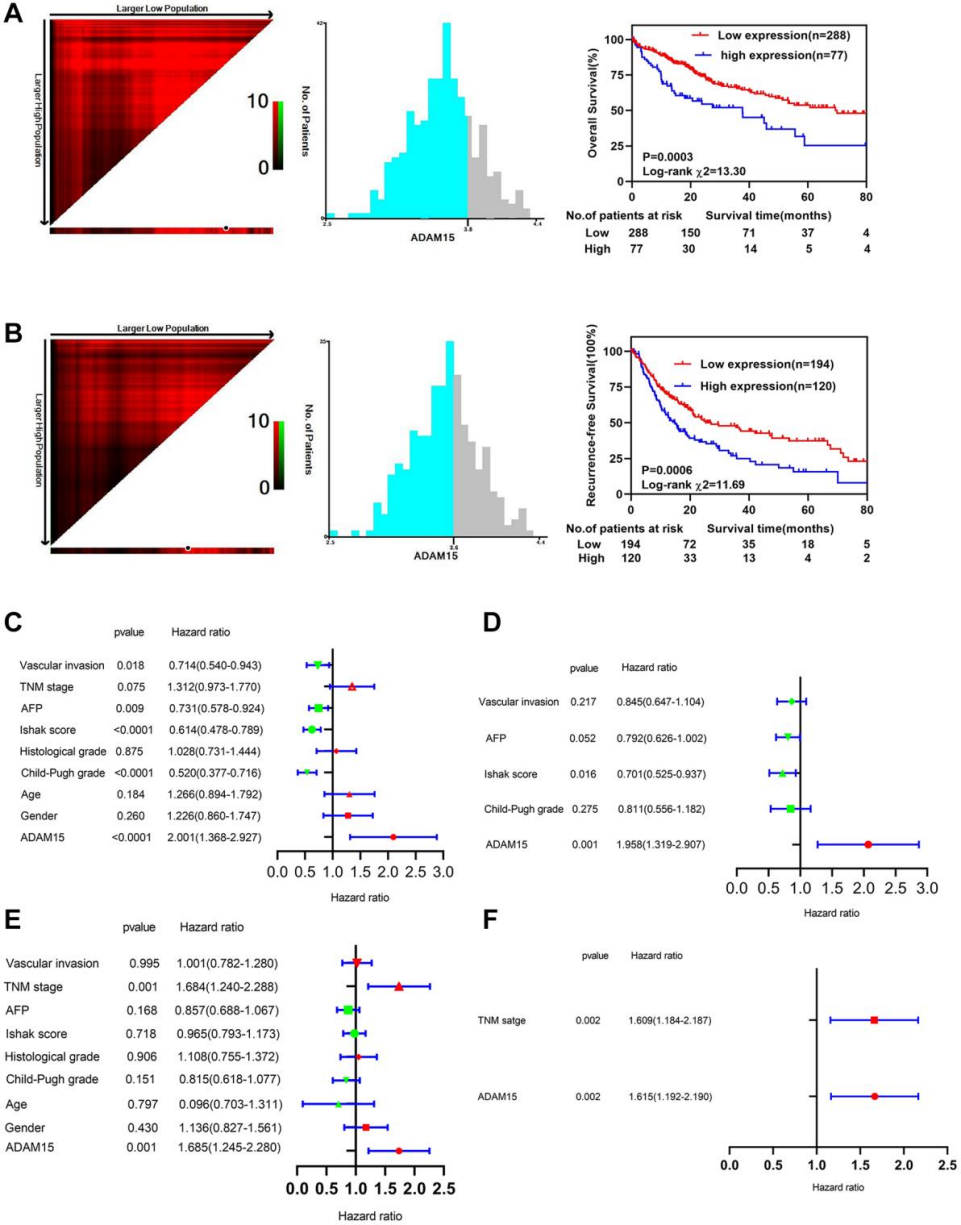
**Survival analysis and prognostic value of ADAM15 between different groups.** (**A**) Kaplan-Meier survival analysis of OS in HCC. (**B**) Kaplan-Meier survival analysis of RFS in HCC. (**C**) Univariate Cox regression analysis of OS in HCC based on TCGA database. (**D**) Multivariate Cox regression analysis of OS in HCC based on TCGA database. (**E**) Univariate Cox regression analysis of RFS in HCC based on TCGA database. (**F**) Multivariate Cox regression analysis of RFS in HCC based on TCGA database.

Then, we performed ROC curve analyses to confirm the diagnostic value of ADAM15 in HCC using the TCGA and GEO databases, the results showed that AUC = 0.9198, *P* < 0.0001 in TCGA (Figure 1H), AUC = 0.8256, *P* < 0.0001 (GSE76427) (Figure 1I) and AUC = 0.7606, *P* < 0.001 (GSE54236) in GEO (Figure 1J). To verify the relationship between ADAM15 expression and clinicopathological features, the results in Table 1 have shown that the level of ADAM15 expression is not related to age, gender, histological grade, Ishak score, alpha-fetoprotein (all *P* > 0.05). However, there was a significant association between ADAM15 expression and recurrence status, living status, Child-Pugh grade and vascular invasion (all *P* < 0.05). Then, the prognostic value of ADAM15 in HCC was estimated by KM curves based on the optimal thresholds calculated by X-tile software. Our findings manifested that overexpression of ADAM15 was connected with poor OS and RFS (all *P* < 0.05) (Figure 2A, 2B). To further verify the effect of ADAM15 expression on the survival of HCC patients, we performed survival analysis by the Kaplan Meier plotter online database, our findings revealed that ADAM15 expression was related to OS (*P* = 0.0074), RFS (*P* = 0.012) and Progress Free Survival (PFS) (*P* = 0.026) (Supplementary Figure 1A–1C), and was not correlated with Disease Free Survival (DSS) (*P* > 0.05) (Supplementary Figure 1D). Moreover, the Cox proportional hazard regression model was adopted to analyze the independent indicators of OS and RFS. In Figure 2C, we found that the level of ADAM15 expression, alpha-fetoprotein, Child-Pugh grade, Ishak score and vascular invasion was significantly correlated with poor OS by univariate analysis (all *P* < 0.05). Among them, only ADAM15 expression and Ishak score were associated with poor OS based on multivariate analysis (all *P* < 0.05) (Figure 2D). Using the same method, we observed that ADAM15 expression and TNM stage were prominently correlated with poor RFS by both univariate analysis and multivariate analysis (all *P* < 0.05) (Figure 2E, 2F).

**Table 1:** The relationships between ADAM15 expression and clinicopathological parameters in HCC.

**Variables**	**ADAM15 expression**		***χ*^2^**	***P***
	**Low (*n* = 288)**	**High (*n* = 77)**		
Gender			1.262	0.261
Male	190 (66.0%)	56 (72.7%)		
Female	98 (34.0%)	21 (27.3%)		
Age			1.058	0.304
≦65	183 (63.5%)	44 (57.1%)		
>65	105 (36.5%)	33 (42.9%)		
Child-Pugh grade			14.525	**0.001**
A	178 (61.8%)	38 (49.4%)		
B-C	22 (7.6%)	0 (0)		
Unknow	88 (30.6%)	39 (50.6%)		
TNM stage			6.000	0.050
I-II	209 (72.6%)	45 (58.4%)		
III-IV	61 (21.2%)	26 (33.8%)		
Unknow	18 (6.3%)	6 (7.8%)		
Histologic grade			4.133	0.127
G1-G2	189 (65.6%)	41 (63.0%)		
G3-G4	95 (33.0%)	35 (35.6%)		
Unknow	4 (1.4%)	1 (1.4%)		
Vascular invasion			7.758	**0.021**
No	171 (59.4%)	34 (44.2%)		
Yes	81 (28.1%)	25 (32.5%)		
Unknow	36 (12.5%)	18 (23.4%)		
Alpha fetoprotein			3.575	0.167
Negative	123 (42.7%)	24 (31.2%)		
Positive	99 (34.4%)	30 (39.0%)		
Unknow	66 (22.9%)	23 (29.9%)		
Ishak score			1.498	0.473
0–4	105 (36.5%)	27 (35.1%)		
5–6	64 (22.2%)	13 (16.9%)		
Unknow	119 (41.3%)	37 (48.1%)		
Living status			8.027	**0.005**
Alive	196 (68.1%)	39 (50.6%)		
Dead	92 (31.9%)	38 (49.4%)		
Recurrence status			9.061	**0.011**
No	121 (42.0%)	20 (26.0%)		
Yes	133 (46.2%)	40 (51.9%)		
Unknow	34 (11.8%)	17 (22.1%)		

Statistically significant *p* values are given in bold, *P* < 0.05.

### Validation of the prognostic value of ADAM15 in HCC based on nomograms

**Figure 3:**
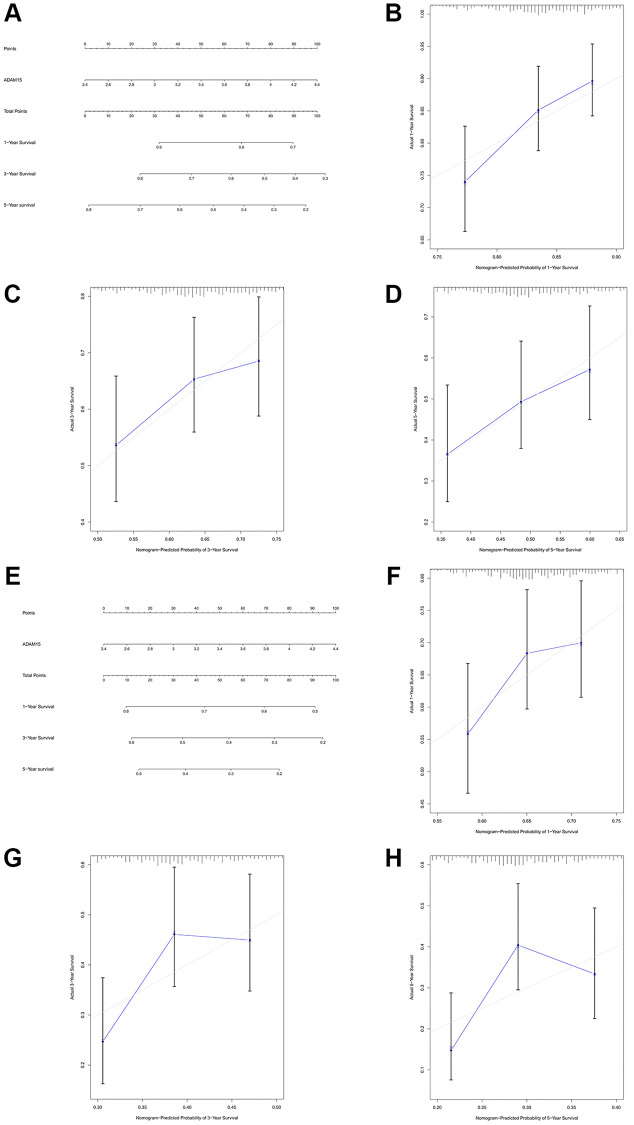
**The prognostic value of ADAM15 was validated by nomograms.** (**A**) The nomogram of OS. (**B**) The 1-year survival rate of OS. (**C**) The 3-year survival rate of OS. (**D**) The 5-year survival rate of OS. (**E**) The nomogram of RFS. (**F**) The 1-year survival rate of RFS. (**G**) The 3-year survival rate of RFS. (**H**) The 5-year survival rate of RFS.

To further manifest the prognostic value of ADAM15 in HCC, we constructed nomograms based on ADAM15 expression to predict the possibility of 1-year, 3-year and 5-year OS and RFS. As shown in Figure 3A and Figure 3E, we could calculate 1-year, 3-year and 5-year survival rates of HCC patients based on ADAM15 expression. The indication of calibration curve matches well (Figure 3B–3D and Figure 3F–3H). The C-index of a nomogram for OS prediction was 0.596 (95% CI: 0.569–0.624), and the C-index of a nomogram for RFS prediction was 0.557 (95% CI: 0.534–0.581).

### ADAM15 expression correlated with immune infiltration, stromal scores and immune scores in HCC

**Figure 4:**
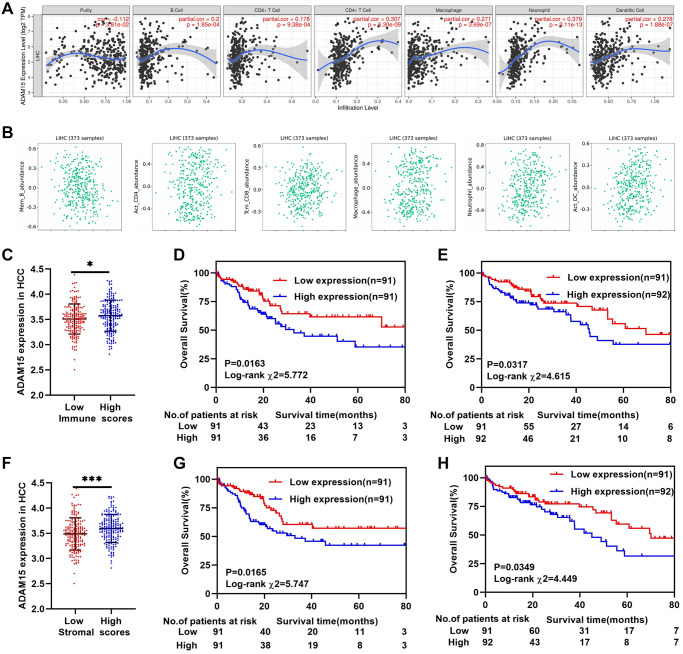
**ADAM15 expression associated with immune infiltration in HCC.** (**A**, **B**) The relationship between ADAM15 expression and immune infiltration cells confirmed by TIMER and TISIDB databases. (**C**) ADAM15 is highly expressed in the high immune score group based on the TCGA database. (**D**) Kaplan-Meier survival analysis of OS in the low immune score group of HCC based on the TCGA database. (**E**) Kaplan-Meier survival analysis of OS in the high immune score group of HCC based on the TCGA database. (**F**) ADAM15 is highly expressed in high stromal score group based on the TCGA database. (**G**) Kaplan-Meier survival analysis of OS in the low stromal score group of HCC based on the TCGA database. (**H**) Kaplan-Meier survival analysis of OS in the high stromal score group of HCC based on the TCGA database.

Tumor-infiltrating lymphocytes and tumor immune microenvironment can affect the survival time of patients in different types of cancers [28]. As shown in Figure 4A, ADAM15 expression was negatively associated with tumor purity (cor = –0.112, *P* = 3.81e-02), and was positively correlated with B cells (cor = 0.2, *P* = 1.85e-04), CD8+T cells (cor = 0.178, *P* = 9.38e-04), CD4+T cells (cor = 0.307, *P* = 6.30e-09), Macrophages (cor = 0.271, *P* = 3.69e-07), Neutrophils (cor = 0.379, *P* = 3.11e-13) and Dendritic cells (cor = 0.278, *P* = 1.88e-07), of which ADAM15 had the strongest correlation with Neutrophils. Then, we also demonstrated the connection between ADAM15 and immune cell infiltration via the ESTIMATE algorithm according to the TCGA database. The results have revealed that ADAM15 is positively correlated with central memory CD8 T cells (rho = 0.122, *P* = 0.0188), activated CD4 T cells (rho = 0.136, *P* = 0.00854), macrophage cells (rho = 0.194, *P* = 0.00017), neutrophils (rho = 0.236, *P* = 4.44e-06) and activated dendritic cells (rho = 0.243, *P* = 2.11e-06) (Figure 4B). The ESTIMATE algorithm was also used to estimate immune and stromal scores in HCC. The results have shown that immune scores of HCC are ranged from -1209.16 to 2934.36, and stromal scores are distributed from –1741.56 to 1195.07. We further calculated the association of ADAM15 expression with immune scores and stromal scores. 365 patients were separated into the low group and high group based on the median of their scores. Our findings indicated that patients with high immune scores or stromal scores had higher ADAM15 mRNA expression than those with low immune or stromal scores (*P* < 0.05) (Figure 4C, 4F). At the same time, we also assessed the prognostic value of ADAM15 at different immune scores or stromal scores for HCC. As shown in Figure 4D, 4E, there was a positive correlation between ADAM15 high expression and poor OS of HCC in both low and high immune scores (*P* < 0.05). Consistent with the above results, ADAM15 high expression was related to poor OS of HCC in both low and high stromal scores (*P* < 0.05) (Figure 4G, 4H). In addition, we further verified whether there were differences in ADAM15 expression in different immune cells between HCC tissues and paired normal tissues. Based on the analysis of GEPIA database, our observed that ADAM15 expression was markedly different in CD4 *T* cells, NK cells, Monocytes, Macrophage M0, Macrophage M1, Macrophage M2, B naive cells, CD8 T cells, Dendritic cells and Neutrophils of HCC tissues (*P* < 0.05) (Supplementary Figure 2A, 2C). There were statistical differences in the expression of ADAM15 in Monocyte (F = 26.06, *P* = 5.03e-7), B naive cells (F = 6.59, *P* = 0.01), Macrophages M0 (F = 29.11, *P* = 1.15e-7), Macrophages M2 (F = 12.73, *P* = 4.02e-4) and Neutrophils (F = 6.24, *P* = 0.01) (Supplementary Figure 2B, 2D). These results indicated that ADAM15 was highly expressed not only in HCC tissues, but also in immune cells of HCC tissues. Collectively, this gave us reason to believe that ADAM15 increased the proliferation, migration and invasion capacities, at least in part by modulating tumor immune infiltration. However, *in vivo* and *in vitro* experiments are needed to verify the above hypothesis.

### The relationship between ADAM15 expression and immune cell markers

**Figure 5:**
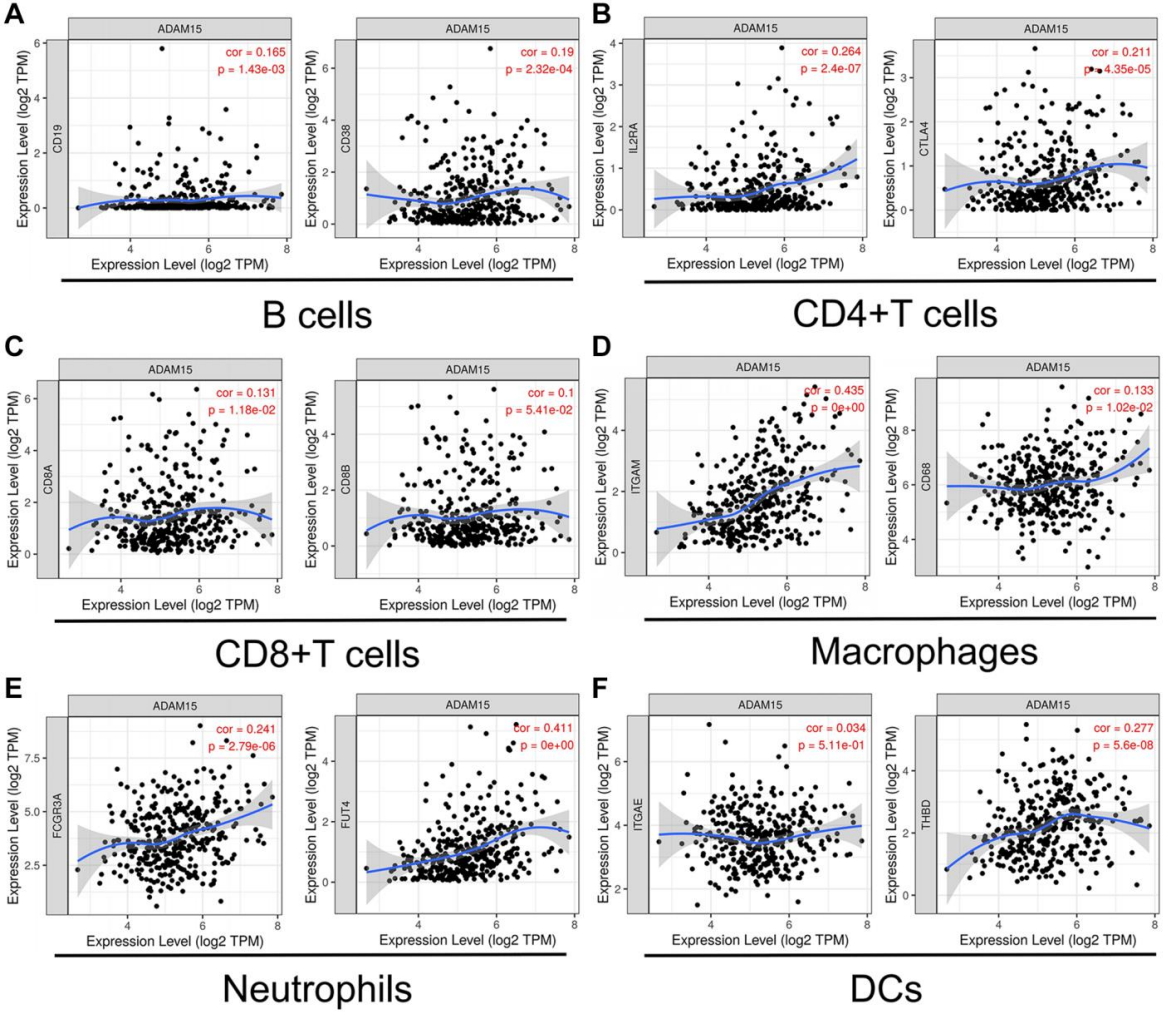
**ADAM15 expression was correlated with markers of immune cells.** (**A**) B cell markers, CD19 and CD38. (**B**) CD4+T cell markers, IL2RA and CTLA4. (**C**) CD8+T cell, CD8A and CD8B. (**D**) Macrophage markers, ITGAM and CD68. (**E**) Neutrophil markers, FCGR3A and FUT4. (**F**) DC markers, ITGAE and THBD.

Next, we explored the relationships between ADAM15 expression and surface markers of immune cells in HCC using the TIMER database. Our findings have demonstrated that ADAM15 expression is positively connected with the surface markers of B cells (CD19: cor = 0.165, *P* = 1.43e-03; CD38: cor = 0.19, *P* = 2.32e-04), CD4+T cells (IL2RA: cor = 0.264, *P* = 2.4e-07; CTLA4: cor = 0.211, *P* = 4.35e-05), CD8+T cells (CD8A: cor = 0.131, *P* = 1.18e-02; CD8B: cor = 0.1, *P* = 5.41e-02), Macrophages (ITGAM: cor = 0.435, *P* = 0; CD68: cor = 0.133, *P* = 1.02e-02) and Neutrophils (FCGR3A: cor = 0.241, *P* = 2.79e-06; FUT4: cor = 0.411, *P* = 0), Dendritic cells (DCs) (ITGAE: cor = 0.034, *P* = 5.11e-01; THBD: cor = 0.277, *P* = 5.6e-08) (Figure 5A–5F). However, the level of gene expression in tumor cells was negatively associated with tumor purity, the correlation analysis between ADAM15 expression and immune cell markers was adjusted by tumor purity (Table 2). There were positive relationships between ADAM15 expression and markers of Tregs (IL2RA: cor = 0.264, *P* = 2.40e-07; INFRSF18: cor = 0.340, *P* = 1.80e-11), Macrophages (CD68: cor = 0.133, *P* = 1.02e-02; ITGAM: cor = 0.435, *P* = 0), TAMs (CD80: cor = 0.267, *P* = 1.71e-07; CD86: cor = 0.253, *P* = 8.30e-07), NKs (XCL1, cor = 0.124, *P* = 1.66e-02; CD7: cor = 0.198, *P* = 1.25e-04), Th2 (IL17RB: cor = 0.142, *P* = 6.26e-03; STAT6: cor = 0.266; *P* = 1.99e-07), Tfh (CXCR5: cor = 0.129, *P* = 1.30e-02; CD84: cor = 0.233, *P* = 5.61e-06) and T cell exhaustion (PD-1: cor = 0.181, *P* = 4.61e-04; CTLA4: cor = 0.211, *P* = 4.35e-05; TIM-3: cor = 0.307, *P* = 1.77e-09) (Table 2). Therefore, the above findings indicated that ADAM15 expression was related to immune cell infiltration in HCC.

**Table 2:** Correlation between ADAM15 expression and immune cell surface markers.

**Cell type**	**Gene markers**	**LIHC**			
		**None**		**Purity**	
		**Cor**	***P***	**Cor**	***P***
B cells	CD19	1.43e-03	0.165	0.012	5.73e-02
	CD38	0.190	**2.32e-04**	0.154	**4.09e-03**
CD8+T	CD8A	0.131	**1.18e-02**	0.063	2.43e-01
	CD8B	0.100	5.41e-02	0.029	5.90e-01
Treg	CD25/IL2RA	0.264	**2.40e-07**	0.230	**1.53e-05**
	GITR/TNFRSF18	0.340	**1.80e-11**	0.313	**2.95e-09**
M	CD68	0.133	**1.02e-02**	0.072	1.80e-01
	CD11b/ITGAM	0.435	**0**	0.422	**2.58e-16**
M1	NOS2	0.201	**1.00e-04**	0.189	**4.31e-04**
	IFNGR1	0.015	7.77e-01	–0.014	8.02e-01
M2	ARG1	–0.015	7.74e-01	–0.008	8.88e-01
	MRC1	0.128	**1.40e-02**	0.009	9.66e-02
TAM	CD80	0.267	**1.71e-07**	0.246	**3.66e-06**
	CD86	0.253	**8.30e-07**	0.204	**1.34e-04**
Monocyte	CD14	–0.030	5.66e-01	–0.023	6.76e-01
	CD163	0.151	**3.60e-03**	0.083	1.26e-01
NK	XCL1	0.124	**1.66e-02**	0.102	5.68e-02
	CD7	0.198	**1.25e-04**	0.146	**6.55e-03**
Neutrophil	CD15/FUT4	0.411	**0**	0.381	**2.34e-13**
	MPO	0.074	1.55e-01	0.012	8.30e-01
DC	CD103/ITGAE	0.034	5.11e-01	0.007	8.96e-01
	CD141/THBD	0.277	**5.60e-08**	0.242	**5.36e-06**
Th1	IL12RB2	–0.004	9.40e-01	–0.046	3.90e-01
	CXCR3	0.235	**5.01e-06**	0.183	**6.33e-04**
Th2	IL17RB	0.142	**6.26e-03**	0.145	**6.98e-03**
	STAT6	0.266	**1.99e-07**	0.262	**7.92e-07**
Th9	TGFBR2	–0.033	5.27e-01	–0.066	2.18e-01
	PU.1/SPI1	0.319	**3.93e-10**	0.277	**1.78e-07**
Th17	IL21R	0.296	**6.51e-09**	0.253	**1.92e-06**
	IL6RA/IL6R	–0.009	8.60e-01	0.033	5.43e-01
Th22	CCR10	0.295	**7.12e-09**	0.277	**1.64e-07**
	AHR	–0.016	7.55e-01	–0.034	5.28e-01
Tfh	CXCR5	0.129	**1.30e-02**	0.081	1.36e-01
	CD84	0.233	**5.61e-06**	0.207	**1.05e-04**
T cell exhaustion	PD-1/PDCD1	0.181	**4.61e-04**	0.127	**1.80e-02**
	CTLA4	0.211	**4.35e-05**	0.168	**1.78e-03**
	TIM-3/HAVCR2	0.307	**1.77e-09**	0.276	**1.97e-07**

Abbreviations: LIHC: hepatocellular carcinoma; M: macrophage; M1: macrophage M1: M2, macrophage M2; TAMs: tumor-associated macrophage; NK: natural killer cell; DC: dendritic cell; Th: T helper cell; Tfh: Follicular help cell; Treg: regulatory *T* cell; Cor: *R* value of Spearman’s correlation; None: correlation without adjustment; Purity: correlation adjustment by purity. *P* < 0.05 is considered statistical significance with black bold font.

### The association of ADAM15 expression with immune checkpoint genes for liver cancer

**Figure 6:**
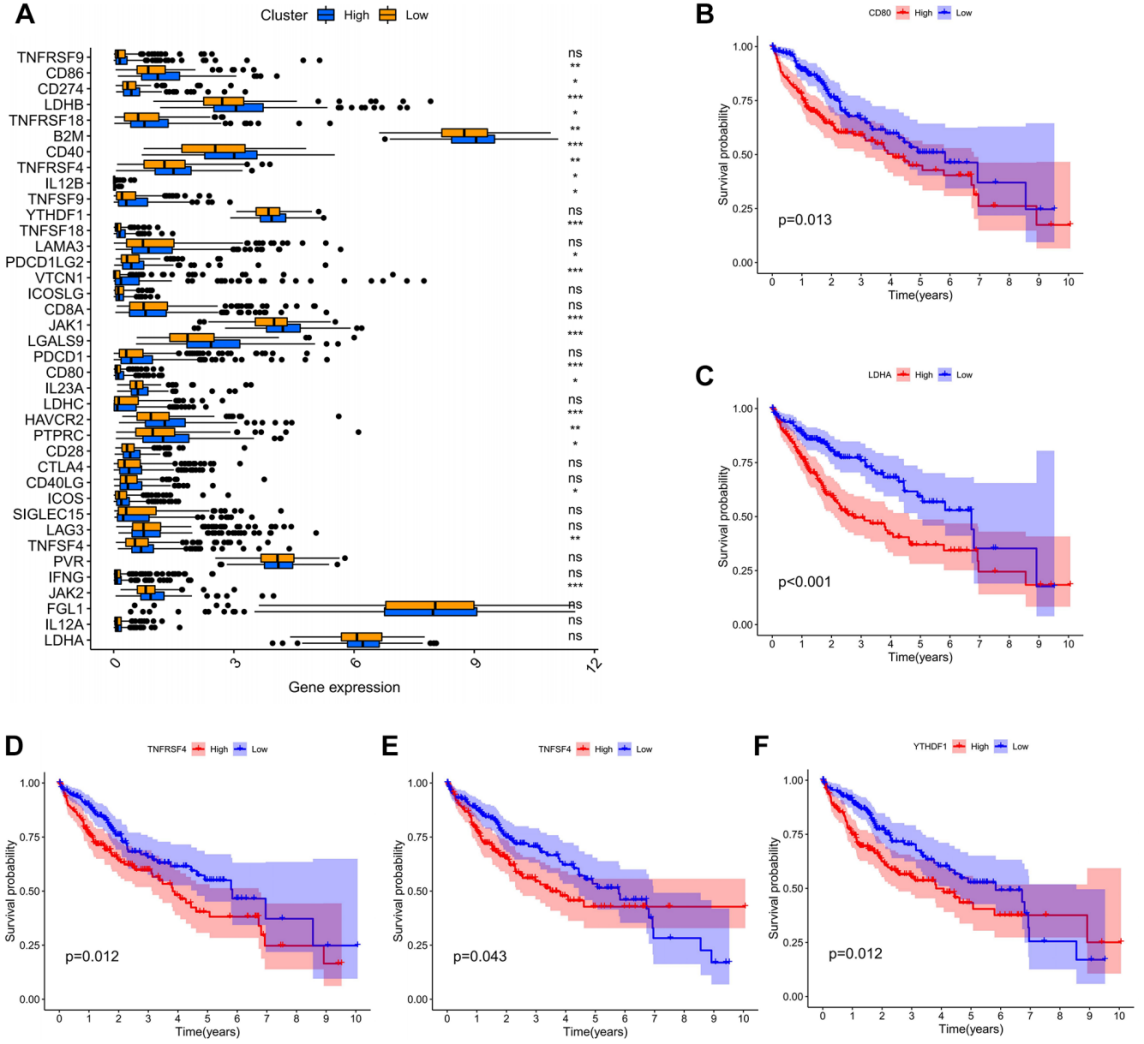
**The association of ADAM15 expression with immune checkpoint genes.** (**A**) The differential expression of immune checkpoint genes between the high and low expression group. (**B**) The survival analysis of CD80. (**B**) The survival analysis of LDHA. (**C**) The survival analysis of TNFRSF4. (**D**) The survival analysis of TNFSF4. (**E**) The survival analysis of TNFSF4. (**F**) The survival analysis of YTHDF1.

At present, many genes are involved in the immune response and are considered to be the immune checkpoint genes. We investigated the expression of 38 immune checkpoint genes between the high and low expression group, and performed OS of the above differentially expressed genes (DEGs). The results have shown that CD86, CD274, LDHB, TNFRSF18, B2M, CD40, TNFRSF4, IL12B, TNFSF9, TNFSF18, PDCDILG2, VTCN1, TAK1, LGALS9, CD80, IL23A, HAVCR2, PTPRC, CD28, ICOS, TNFSF4 and JAK2 expression was statistically different between the high and low group (*P* < 0.05) (Figure 6A). Then, we further explored OS of DEGs in the high and low group, and only the survival analysis of CD80, LDHA, TNFRSF4, TNFSF4 and YTHDF1 showed statistical significance (*P* < 0.05) (Figure 6B–6F). The above five immune checkpoint genes could be utilized as potential targets for immunotherapy of HCC.

### ADAM15 expression affected HCC cells proliferation, invasion and migration

**Figure 7:**
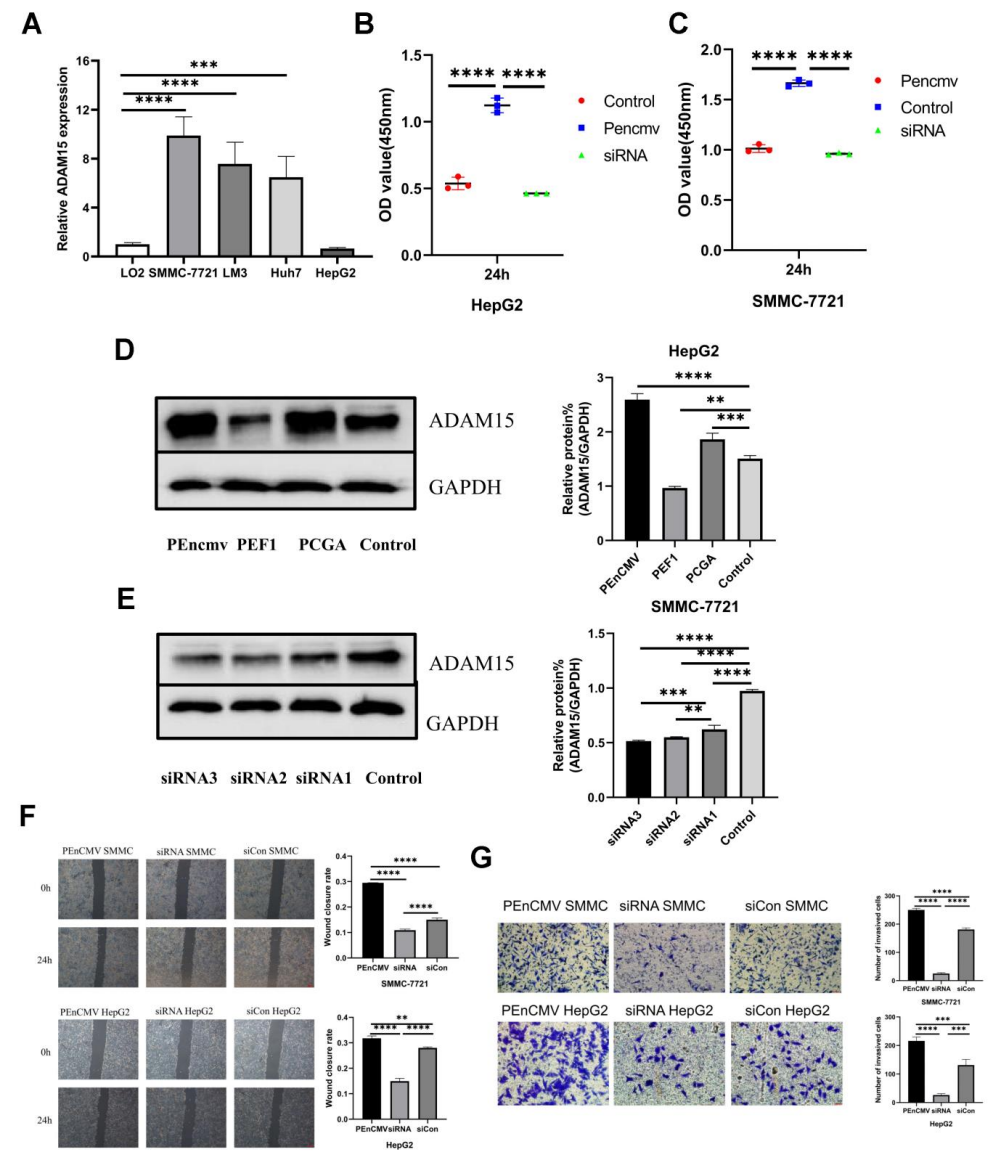
**The level of ADAM15 expression was associated with the proliferation, migration and invasion of HCC cells.** (**A**) Comparison of ADAM15 expression in HCC cells and liver cells. (**B**, **C**) Cell proliferation ability of HepG2 and SMMC-7721 cell in siCon group, siADAM15 group and ADAM15 overexpression group was examined by CCK8 assay. (**D**) Overexpression of ADAM15 was evaluated by western blot in HepG2 cell. (**E**) ADAM15 knockdown was assessed by western blot in SMMC-7721 cell. (**F**) Cell migration ability of HepG2 and SMMC-7721 cell in siCon group, siADAM15 group and ADAM15 overexpression group was examined by a wound-healing method. Magnification, x40. Scale bar: 200 μm (**G**) Cell invasion ability of HepG2 and SMMC-7721 cell in siCon group, siADAM15 group and ADAM15 overexpression group was evaluated by transwell assay. Magnification, x200. Scale bar: 50 μm.

To further explore the biological role of ADAM15 in HCC, we performed transfection experiments *in vitro*. Our results indicated that ADAM15 was highly expressed in SMMC-7721 cells, and was down-regulated in HepG2 cells (Figure 7A). Therefore, SMMC-7721 cells were transfected with siRNAs, HepG2 cells were transfected with ADAM15 overexpression plasmid, and the result of western blot showed that siRNA3 and PEnCMV had the best effect (Figure 7D, 7E). CCK8 assays, wound healing and transwell assays were performed to investigate whether ADAM15 affected the proliferation, migration and invasion of HCC cells, the results have revealed that siADAM15-infected cells (SMMC-7721) significantly suppress the proliferation, invasion and migration of HCC cells. However, HepG2 cell was transfected with PEnCMV plasmid exhibited a conspicuous increase in the proliferation, invasion and migration of HCC cells compared with the siCon-transfected cells (Figure 7B, 7C, 7F, 7G).

### ADAM15 expression correlated with the apoptosis of HCC cells

**Figure 8:**
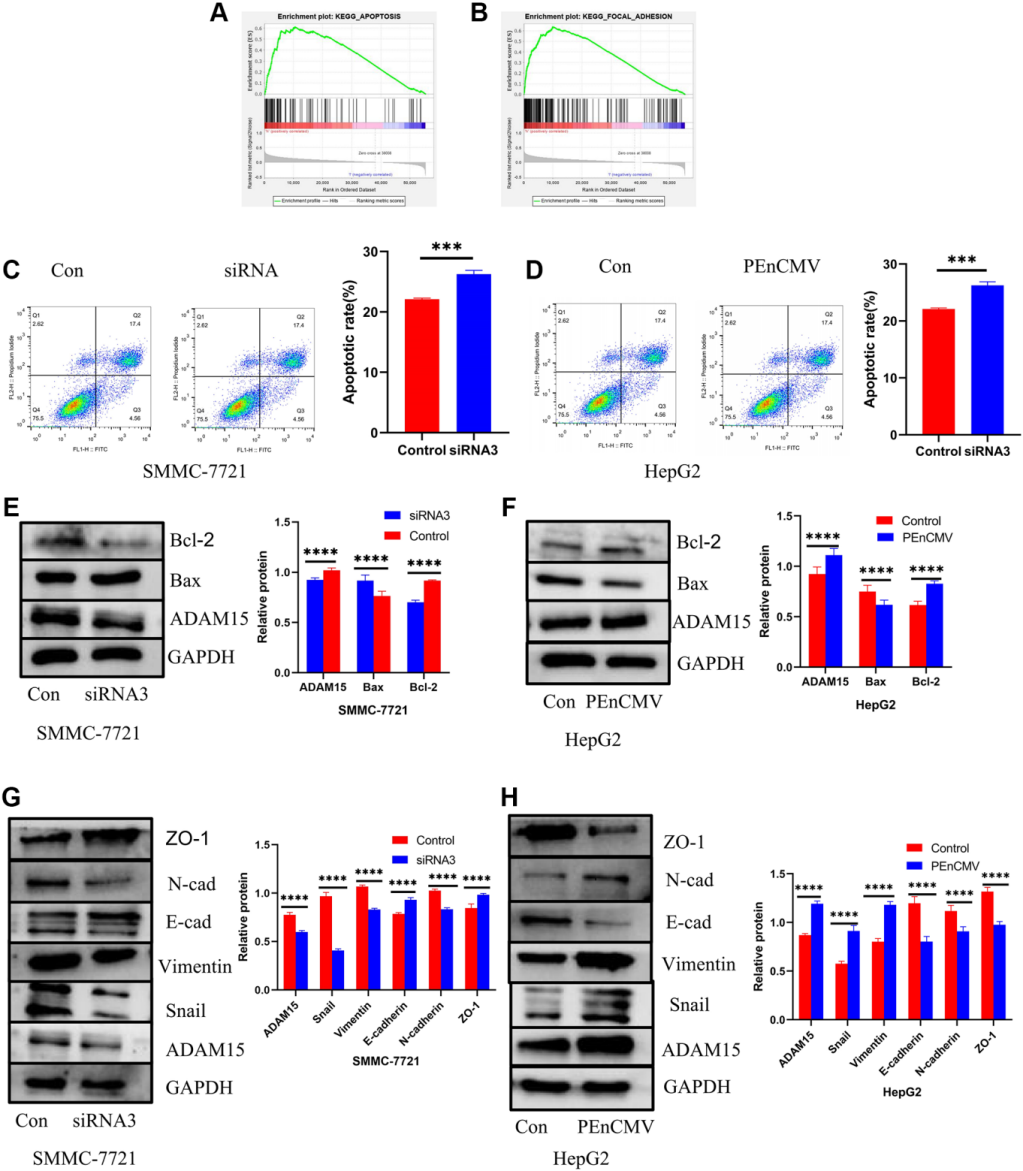
**Impacts of ADAM15 on the expression of apoptosis-related proteins and EMT-related proteins.** (**A**) Functional enrichment analyses indicated that overexpression of ADAM15 was associated with apoptosis. (**B**) Functional enrichment analyses indicated that overexpression of ADAM15 was related to focal adhesion. (**C**) Flow cytometry analysis indicated that the siRNA-transfected in SMMC-7721 cells significantly induced apoptosis. (**D**) Flow cytometry analysis indicated that the overexpression of ADAM15 in HepG2 cells significantly suppressed apoptosis. (**E**) Comparison of apoptosis-related protein expression between the control group and siRNA3 group in SMMC-7721 cells. (**F**) Comparison of apoptosis-related protein expression between the control group and overexpression of ADAM15 group in HepG2 cell. (**G**) Comparison of EMT-related protein expression between the control group and siRNA3 group in SMMC-7721 cells. (**H**) Comparison of EMT-related protein expression between the control group and overexpression of ADAM15 group in HepG2 cells.

Functional enrichment analyses indicated that overexpression of ADAM15 was associated with apoptosis and focal adhesion (Figure 8A, 8B). Therefore, we performed flow cytometry analysis on HCC cells with Annexin V/PI. The siRNA-transfected HCC cells significantly induced apoptosis, and overexpression of ADAM15 remarkably suppressed the apoptosis of HCC cells (Figure 8C, 8D). To further dissect the underlying mechanisms of ADAM15 knockdown induced apoptosis in HCC cells, we performed western blot to examine the expression of apoptosis-related proteins (Bax and Bcl-2) of HCC cells with ADAM15 knockdown and ADAM15 overexpression. Western blot analyses indicated that the level of Bax protein was significantly decreased, and the level of Bcl-2 protein was markedly increased in the siADAM15 SMMC-7721 cell compared with the siCon-transfected cell (Figure 8E). In line with the above findings, the level of Bax protein was significantly increased, and the level of Bcl-2 protein was markedly decreased in the PEnCMV-transfected HepG2 cell compared with the siCon-transfected cell (Figure 8F).

### ADAM15 knockdown inhibits HCC cells migration and invasion by regulating EMT

As we known, ADAM15 served a critical role in the regulation of cell adhesion and cell vitality, and functional enrichment analyses showed that ADAM15 expression correlated with cell adhesion. Therefore, we investigated the relationship between ADAM15 expression and epithelial-mesenchymal transformation (EMT) in HCC cells. Western blot analyses revealed that the level of ZO-1, E-cadherin was increased, and the level of Vimentin, N-cadherin and Snail was decreased in siADAM15 SMMC-7721 cell compared with the siCon-transfected cell (Figure 8G). On the contrary, the level of ZO-1, E-cadherin was decreased, and the level of Vimentin, N-cadherin and Snail was elevated in PEnCMV-transfected SMMC-7721 cell compared with the siCon-transfected cell (Figure 8H). The above results manifested that ADAM15 served a crucial role in the invasion and metastasis of HCC cells.

## DISCUSSION

Hepatocellular carcinoma, as the main pathologic type of liver cancer, has high morbidity and mortality in the world [29]. The prognosis for HCC is very poor, as only 5% to 15% of patients are eligible for surgical treatment [1]. So, it is particularly important to investigate new therapeutic targets for HCC.

ADAM15, as one member of the ADAM gene family, can encode transmembrane metalloproteinase-disintegrins [30], and participates in diverse physiological and pathological processes by their proteolysis and adhesion activity [31]. Some studies have demonstrated that ADAM15 is overexpressed in many tumors, such as prostate cancer, melanoma and breast cancer [12, 15]. In the present study, the level of ADAM15 expression was higher in HCC samples than that in adjacent noncancerous samples based on the TCGA and GEO analyses, and overexpression of ADAM15 came with worse OS and RFS. Consistent with the above results, the results of RT-qPCR, IHC and Western blot also indicated that ADAM15 was up-regulated in HCC tissues and cells compared with normal tissues and liver cells. Then, we further demonstrated the role of ADAM15 in HCC *in vitro* experiments, CCK-8 assays revealed that ADAM15 knockdown significantly inhibited the viability of HCC cells, and wound-healing and transwell invasion assays demonstrated that ADAM15 knockdown remarkably reduced cell migratory and invasive capability compared with the control group, and overexpression of ADAM15 significantly enhanced cell migratory and invasive capability. The above results have shown that overexpression of ADAM15 serves an important role in hepatocarcinogenesis and metastasis. At the same time, our results demonstrated that ADAM15 expression was closely related to tumor immune infiltration via bioinformatics analysis, but *in vitro* and *in vivo* experiments were needed to dissect the role of ADAM15 in tumor immunity.

As we known, signal pathways play a crucial role in tumorigenesis. To further investigate the underlying mechanism of ADAM15 affecting HCC, we performed GSEA functional enrichment analyses, the results have indicated that “KEGG_APOPTOSIS” and “KEGG_FOCAL_ADHESION” are significantly correlated with ADAM15 high expression. Apoptosis, as an order and orchestrated cellular process, plays a crucial role in the physiological and pathological processes, including tumorigenesis [32]. Flow cytometry analysis revealed that ADAM15 knockdown induced apoptosis of HCC cells, while overexpression of ADAM15 inhibited apoptosis. Meanwhile, the results of western blot indicated that ADAM15 knockdown in SMMC-7721 cell was companied by the decrease of Bcl-2 protein expression and the increase of Bax protein expression, and overexpression of ADAM15 in HepG2 cell would induce the increase of Bcl-2 protein expression and the decrease of Bax protein expression. Numerous researches indicated that EMT was related to tumor initiation, tumor development, tumor cell migration, invasion and metastasis [33, 34]. To further elucidate the role of ADAM15 in HCC cell migration and invasion, we confirmed that a reduction in N-cadherin, Vimentin, Snail expression, and an elevation in ZO-1, E-cadherin expression in the ADAM15 knockdown group, whereas overexpression of ADAM15 had the opposite effect on the expression of N-cadherin, Vimentin, E-cadherin, ZO-1 and Snail. Collectively, ADAM15 was involved in the occurrence, development and metastasis of HCC through influencing apoptosis and EMT. However, the underlying molecular mechanism of ADAM15 regulating apoptosis and EMT was needed further research.

In conclusion, our results demonstrated that the level of ADAM15 expression was higher in HCC tissues and cells than that in paired noncancerous tissues and liver cells. Overexpression of ADAM15 was connected with poor OS and RFS. There was a relationship between ADAM15 and immune infiltration. ADAM15 knockdown can significantly inhibit HCC cell proliferation, migration and invasion. Furthermore, our findings further confirmed that ADAM15 served a crucial role in the progress of HCC at least in part by suppressing apoptosis and increasing EMT. Therefore, ADAM15 may be a potential target for HCC treatment.

## Supplementary Materials

Supplementary Figures

Supplementary Tables
