# Supplementary material for: ADAM15 correlates with prognosis, immune infiltration and apoptosis in hepatocellular carcinoma
Source: Aging (Albany NY). 2021 Aug 23;13(16):20395–417. doi: 10.18632/aging.203425 (PMC8436918; doi:10.18632/aging.203425)
Supplement: Supplementary Figures [file aging-13-203425-s001.pdf]

SUPPLEMENTARY FIGURES

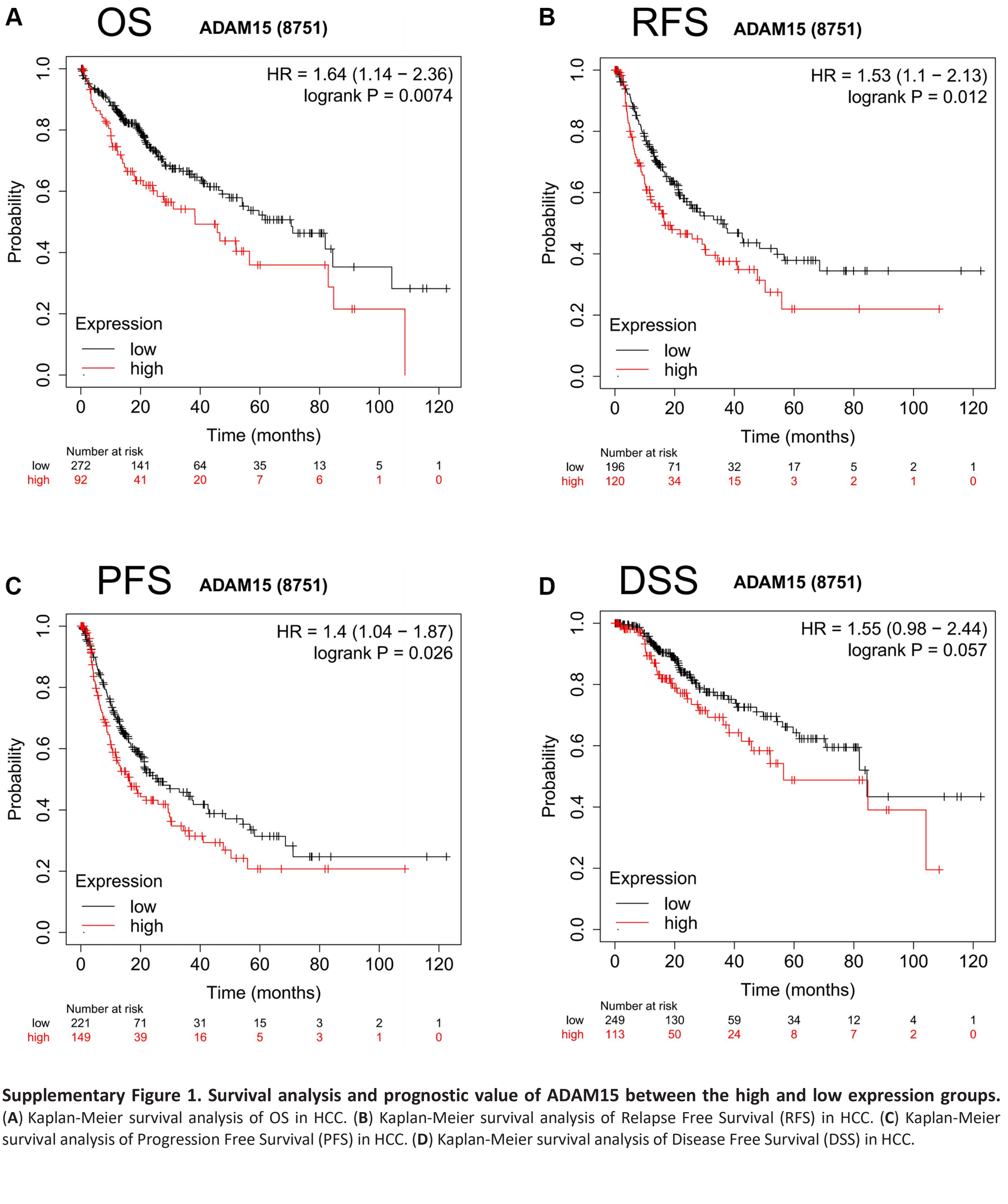

Supplementary Figure 1. Survival analysis and prognostic value of ADAM15 between the high and low expression groups. (**A**) Kaplan-Meier survival analysis of OS in HCC. (**B**) Kaplan-Meier survival analysis of Relapse Free Survival (RFS) in HCC. (**C**) Kaplan-Meier survival analysis of Progression Free Survival (PFS) in HCC. (**D**) Kaplan-Meier survival analysis of Disease Free Survival (DSS) in HCC.

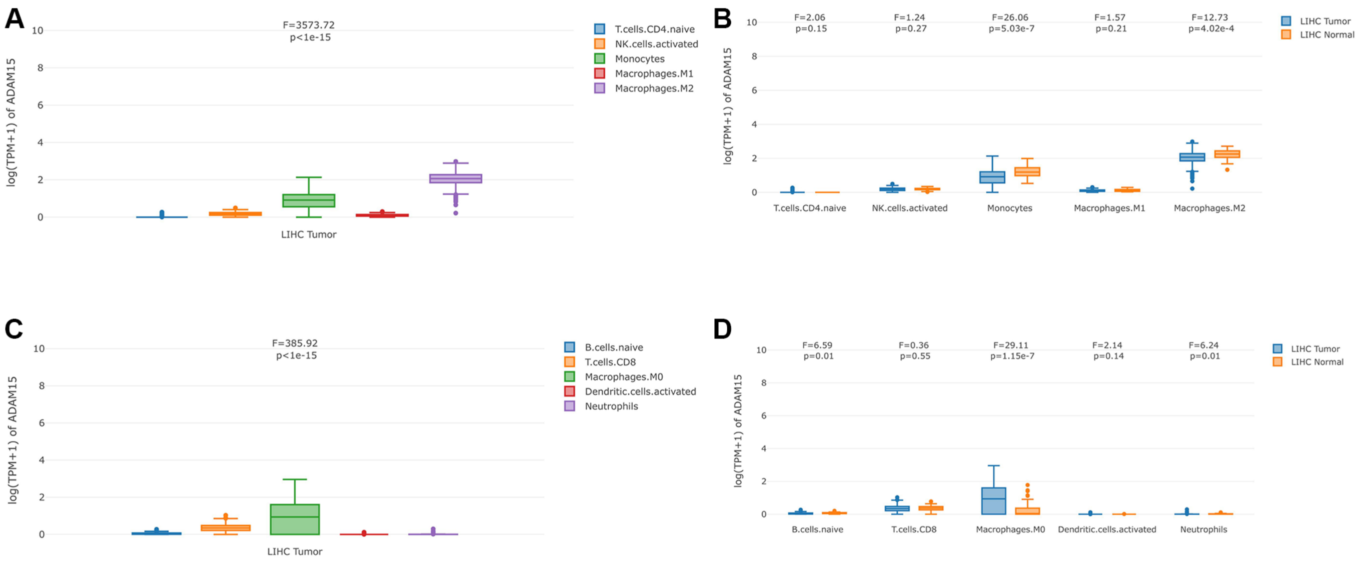

Supplementary Figure 2. The expression of ADAM15 in different immune infiltration cells between liver cancer tissues and paired normal tissues. (**A**) The expression of ADAM15 in T cells CD4 naive, NK cells activated, Monocytes, Macrophages M1 and Macrophages M2 in liver cancer tissues. (**B**) The expression of ADAM15 in T cells CD4 naive, NK cells activated, Monocytes, Macrophages M1 and Macrophages M2 between liver cancer tissues and corresponding normal tissues. (**C**) The expression of ADAM15 in B naive cells, CD8 T cells, Macrophages M0, Dendritic cells and Neutrophils in liver cancer tissues. (**D**) The expression of ADAM15 in B naive cells, CD8 T cells, Macrophages M0, Dendritic cells and Neutrophils between liver cancer tissues and paired normal tissues.
