# Supplementary material for: ADAM15 correlates with prognosis, immune infiltration and apoptosis in hepatocellular carcinoma
Source: Aging (Albany NY). 2021 Aug 23;13(16):20395–417. doi: 10.18632/aging.203425 (PMC8436918; doi:10.18632/aging.203425)
Supplement: Supplementary Tables [file aging-13-203425-s002.pdf]

SUPPLEMENTARY TABLES

Supplementary Table 1. The list of immune checkpoint genes.

YTHDF1
VTCN1
TNFSF9
TNFSF4
TNFSF18
TNFRSF9
TNFRSF4
TNFRSF18
SIGLEC15
PVR
PTPRC
PDCD1LG2
PDCD1
CD274
LGALS9
LDHC
LDHB
LDHA
LAMA3
LAG3
JAK2
JAK1
IL23A
IL12B
IL12A
IFNG
ICOSLG
ICOS
HAVCR2
FGL1
CTLA4
CD8A
CD86
CD80
CD40LG
CD40
CD28
B2M

Supplementary Table 2. The list of siRNA and overexpression plasmid sequences.

Gene	Forward 5′-3′
siRNA sequences	
siRNA1-ADAM15	CCCGAGTATTAGCTGTCTGG
siRNA2-ADAM15	GAATGTACGAGTGGCACTAG
siRNA3-ADAM15	AGTGCCACTCGTACATTCAG
overexpression plasmid sequences	
pCAG-ADAM15	GACTACAAAGACCATGACGGTGATTATAAAGATCATGACATCGACTACAAGGATGACGATGACAAG
pEF1-ADAM15	GACTACAAAGACCATGACGGTGATTATAAAGATCATGACATCGACTACAAGGATGACGATGACAAG
pEnCMV-ADAM15	GACTACAAAGACCATGACGGTGATTATAAAGATCATGACATCGACTACAAGGATGACGATGACAAG
